# Gene Expression Profile of Cultured Human Coronary Arterial Endothelial Cells Exposed to Serum from Chronic Kidney Disease Patients: Role of *MAPK* Signaling Pathway

**DOI:** 10.3390/ijms26083732

**Published:** 2025-04-15

**Authors:** Angélica Rangel-López, Minerva Mata-Rocha, Oscar Alberto Pérez-González, Ricardo López-Romero, Dulce María López-Sánchez, Sergio Juárez-Méndez, Vanessa Villegas-Ruiz, Alfonso Méndez-Tenorio, Juan Manuel Mejía-Araguré, Oscar Orihuela-Rodríguez, Cleto Álvarez-Aguilar, Abraham Majluf-Cruz, Dante Amato, Sergio Zavala-Vega, Silvia Melchor-Doncel de la Torre, Ramón Paniagua-Sierra, José Arellano-Galindo

**Affiliations:** 1 Unidad de Investigación Médica en Enfermedades Nefrológicas, UMAE Hospital de Especialidades, Centro Médico Nacional (CMN) Siglo XXI (SXXI), Instituto Mexicano del Seguro Social (IMSS), Mexico City 06720, Mexico; angelica.rangell@imss.gob.mx; 2 Laboratorio de Virología, Unidad de Investigación en Enfermedades Infecciosas, Hospital Infantil de México Federico Gómez-Secretaría de Salud (SS), Mexico City 06720, Mexico; melchor.sp@ciencias.unam.mx; 3 Unidad de Investigación Médica en Genética Humana, UMAE Hospital de Pediatría, CMN SXXI IMSS, Mexico City 06720, Mexico; mmata@conahcyt.mx; 4 Laboratorio de Oncología Experimental, Instituto Nacional de Pediatría-SS, Mexico City 04530, Mexico; dr.oscarperez@gmail.com (O.A.P.-G.); sjuarezm@pediatria.gob.mx (S.J.-M.); vanyviru21@gmail.com (V.V.-R.); 5 Unidad de Investigación en Biomedicina y Oncología Genómica, Hospital de Gineco-Pediatría 3A, IMSS, Mexico City 07760, Mexico; ricardolopez007@gmail.com; 6 Centro de Investigación en Enfermedades Infecciosas, Instituto Nacional de Enfermedades Respiratorias, SS, Mexico City 14080, Mexico; dulce.lopez@cieni.org.mx; 7 Laboratorio de Biotecnología y Bioinformática Genómica, ENCB-Instituto Politécnico Nacional, Mexico City 11340, Mexico; amendezt@ipn.mx; 8 Laboratorio de Genómica Funcional del Cáncer, Instituto Nacional de Medicina Genómica, SS, Mexico City 14610, Mexico; jmejia@inmegen.gob.mx; 9 Departamento Clínico de Cardiología-UMAE Hospital de Especialidades, CMN SXXI IMSS, Mexico City 06720, Mexico; oscar.orihuela@imss.gob.mx; 10 Facultad de Ciencias Médicas y Biológicas “Dr. Ignacio Chávez”, Universidad Michoacana de San Nicolas de Hidalgo, Morelia, Michoacán 58020, Mexico; cleto.alvarez@umich.mx; 11 Unidad de Investigación Médica en Hemostasia, Trombosis y Aterogénesis, Hospital General Regional 1, IMSS, Mexico City 03103, Mexico; amajlufc@gmail.com; 12 Facultad de Estudios Superiores Iztacala, Universidad Nacional Autónoma de México, Mexico City 54090, Mexico; dante.amato@unam.mx; 13 Laboratorio Clínico y Banco de Sangre, Instituto Nacional de Neurología y Neurocirugía, SS, Mexico City 14269, Mexico; sergio.zavala@innn.edu.mx

**Keywords:** chronic kidney disease, end-stage renal disease, endothelial cell dysfunction, gene expression profile, human coronary arterial endothelial cells, uremia, uremic toxins, myocardial infarction, microarrays

## Abstract

Patients with end-stage renal disease (ESRD) are at increased risk of cardiovascular disease (CVD), such as myocardial infarction (MI). Uremic toxins and endothelial dysfunction are central to this process. In this exploratory study, we used the Affymetrix GeneChip microarray to investigate the gene expression profile in uremic serum-induced human coronary arterial endothelial cells (HCAECs) from ESRD patients with and without MI (UWI and UWOI groups) as an approach to its underlying mechanism. We also explored which pathways are involved in this process. We found 100 differentially expressed genes (DEGs) among the conditions of interest by supervised principal component analysis and hierarchical cluster analysis. The expressions of four major DEGs were validated by quantitative RT-PCR. Pathway analysis and molecular network were used to analyze the interaction and expression patterns. Ten pathways were identified as the main enriched metabolic pathways according to the transcriptome profiling analysis, which were, among others, positive regulation of inflammatory response, positive regulation of extracellular signal-regulated kinases 1 and 2 (ERK1/2) cascade, cardiac muscle cell development, highlighting positive regulation of mitogen-activated protein kinase (MAPK) activity (*p* = 0.00016). Up- and down-regulation of genes from HCAECs exposed to uremic serum could contribute to increased endothelial dysfunction and CVD in ESRD patients. Our study suggests that inflammation and the ERK-MAPK pathway are highly enriched in kidney disease patients with MI, suggesting their role in ESRD pathology. Further studies and approaches based on MAPK pathway interfering strategies are needed to confirm these data.

## 1. Introduction

Chronic kidney disease (CKD) represents a public health problem because of its increased worldwide prevalence, high rates of morbidity, and overwhelmingly high mortality rate, and it is expected to become the fifth leading cause of death by 2040 [1,2]. This increase seems to be closely related to different causes, among which it should be emphasized that approximately 50% of deaths in patients with CKD, who progress to end-stage renal disease (ESRD) and renal replacement therapy (RRT) is necessary, are related to cardiovascular disease (CVD) [3]. Traditional risk factors are not sufficient to explain the increased risk, so various non-traditional risk factors have been studied, such as retention and accumulation of uremic toxins [4], hyperphosphatemia, and inflammation [5]. In addition, early vascular aging (EVA), defined as a discrepancy between chronological and biological age in the vasculature [6], occurs in these patients and is characterized by chronic low-grade inflammation, muscle atrophy, osteoporosis, frailty, high cardiovascular mortality, increased vessel stiffness, vascular calcification, and endothelial dysfunction [7], all of which are included as independent risk factors for CVD in CKD [8,9,10].

Uremic toxins (UTs) are compounds normally filtered from the blood, excreted by healthy kidneys, and accumulate in the blood and tissues as renal function declines [11,12]. As bioactive compounds, some of these toxins exert adverse biological effects and disrupt normal biological processes through cytotoxicity, disrupted cellular signaling, induction of oxidative stress, and systemic inflammation [13]. UTs are classified by their physicochemical characteristics into three categories: Free water-soluble low molecular weight solutes, protein-bound solutes, and middle molecules [14], and have been implicated in different pathological pathways that could be responsible for complications contributing to the mortality in ESRD patients [15]. It has been estimated that large uremic toxins, which belong to the group of medium molecules and include proinflammatory mediators and other cytokines, constitute 23% of the retained solutes [16]. Studies have shown that some of these toxins induce endothelial dysfunction and leukocyte activation, promote inflammation, thrombosis, and increase vascular oxidative stress in CKD [17]. In addition, investigations in large cohorts of patients with ESRD have classified uremic metabolites as predictive of adverse clinical outcomes, associating them with an increased risk of mortality [18].

Endothelial cells serve as the first barrier that plays a crucial role in maintaining vascular integrity. The endothelial damage is the result of sustained toxic and inflammatory conditions and contributes to the immune dysfunction developing in ESRD patients. The activation of monocytes, macrophages, granulocytes, and endothelial cells coexists with the depletion of natural regulatory T-cells and impaired phagocytic functions of polymorphonuclear leukocytes and monocytes. These alterations seem to be aggravated by dialysis procedures. Endothelial dysfunction can be defined as the shift from physiological to pathological activity in endothelial cells. It is characterized by prothrombotic activity, reduced vasodilatation, and a proinflammatory state [19]. It is observed in small and large caliber arteries in patients with CKD [20,21] and is associated with an increased risk of cardiovascular mortality in CKD [22]. Also, non-traditional risk factors, such as accumulation of uremic toxins, oxidative stress, have been associated with endothelial dysfunction. In addition, the structure and function of vascular smooth muscle cells (VSMC) and the composition of the extracellular matrix play an important role in vessel stiffness (atherosclerosis), which is a process related to changes in the composition of elastin, collagen, and vascular calcification, especially in the tunica media layer [23]. The important role of endothelial cell dysfunction in the pathogenesis of atherosclerosis (ASO), has been demonstrated for several decades and yet should be emphasized. CKD arises from many heterogeneous disease pathways that irreversibly alter kidney function and structure over time. As has been mentioned, approximately half of all deaths among patients with CKD are a direct consequence of CVD, and the severity of CVD and risk of death increase with a decline in kidney function, which is progressive and irreversible. In addition, it has been recognized that the nature and spectrum of CVD in CKD are different in people without kidney disease, including ASO, arteriosclerosis, and calcified arterial and left ventricular dysfunction, among others [12]. Moreover, in ESRD on RRT, it is estimated that cardiovascular mortality is greater than 50% of all deaths [24], of which 20% are caused by myocardial infarction (MI) [25,26]. MI is a serious medical condition produced when blood flow to a part of the heart is reduced for a prolonged period of time, causing damage or death of heart muscle tissue [27] The main cause of MI is atherosclerosis, which is the accumulation of plaques composed of cholesterol, fatty substances, calcium, and fibrin in the walls of the coronary arteries [28]. Over time, these plaques become unstable and rupture. After MI, millions of myocytes die, which activates the innate immune response and triggers leukocyte infiltration into the tissue [29]. Neutrophils and macrophages are responsible for the destruction of the extracellular matrix (ECM). Transforming growth factor-beta (TGF-beta), one of the main cytokines present in the damaged myocardium, promotes the differentiation of BFs into myofibroblasts and, together with macrophages, initiates cardiac tissue remodeling with excessive collagen deposition and the formation of fibrosis and scar tissue [30]. Subsequently, a period of approximately 1–2 months elapses, and the scar matures. The pathological pathways associated with elevated cardiovascular mortality have not been fully elucidated, but the presence of EVA appears to be one of the associated pathways [6]. Some investigations, like Serradell, M. et al. [31], have studied in depth the endothelial damage induced by the uremic environment in an in vitro model through endothelial cell cultures exposed to growth media containing uremic serum from hemodialysis patients, which showed morphological alterations, with irregular shape and heterogeneous size, the abundant presence of vacuoles and an increased number of mitotic cells [31]. Also, they showed increased proliferation, evidenced by morphological analysis, cell cycle assessment by flow cytometry, and activation of mitogen-activated protein kinase (MAPK) [31]. In addition, cells cultured under uremic conditions showed signs of inflammation (evidenced by increased expression of vascular cell adhesion molecule 1 (VCAM-1), intracellular adhesion molecule 1 (ICAM-1), and endothelial-leukocyte adhesion molecule (ELAM-1) on the cell surface), the increased presence of these molecules in the cells, and in their soluble form evidencing p38MAPK protein activation [32]. No signs of increased apoptosis were detected despite the accelerated proliferation observed in EC cultures in response to the uremic medium [31]. Furthermore, these cells produced an extracellular matrix with increased tissue factor expression [33], vWF [32], and thrombomodulin [32] while maintaining normal ADAM metallopeptidase with thrombospondin type 1 motif 13 (ADAMTS13) metalloprotease activity [34]. Savira, F. et al. [35] analyzed whether apoptosis signal-regulated kinase apoptosis kinase 1 (ASK1), a regulator of the cellular response to stress, is involved in cardiac hypertrophy and cardiorenal fibrosis induced by indoxyl sulfate (IS) and p-cresol sulfate (PCS) in vitro, and whether inhibition of ASK1 is beneficial in ameliorating these cellular effects. Analyses showed activation of ASK1 and MAPKs (p38MAPK and extracellular signal-regulated kinases 1 and 2 (ERK1/2)), as well as nuclear factor kappa B (NF-κB) by IS and PCS. In addition, inhibitors of ASK1, ornithine aminotransferase 1 and 3 (OAT1/3), ERK1/2, and p38MAPK suppressed all these effects [35]. A very interesting gene expression study was that of Cardinal, H. et al. [36] (which has largely provided the guidelines for the present experimental study), in which the gene expression pattern of human coronary arterial endothelial cells by microarrays showed six genes involved in the regulation of cell cycle progression: (Cyclin dependent kinase 1 (CDK-1), topoisomerase II, PDZ-binding kinase, CDCA1, SDP35 protein, and transcription factor 8 E2F) and two cholesterol efflux system genes (ABCA1 and ABCG1) which were down-regulated in HCAECs exposed to uremic plasma, which have revealed possible mechanisms underlying endothelial dysfunction in patients with ESRD [36]. Recently, investigations have explored signaling pathway-mediated cell-to-cell interactions (CCIs) in the injured heart and their biological effects, offering insights into the mechanisms underlying CVD and potential therapeutic strategies [37]. Its important to mention that endothelial activation is at the crossroads of alterations in inflammatory and immune mechanisms developing in patients with CKD. The feedback between inflammation and immune pathways further potentiates pathologic responses at the endothelial level. A more precise knowledge of the basic molecular mechanisms involved in the development of endothelial damage may facilitate the development of more specific therapeutic strategies that could alleviate the profound alterations in the inflammatory and immunocompetence mechanisms in CKD [38]. Of note, one of the key molecular pathways involved in endothelial dysfunction and cardiovascular complications in CKD is the MAPK signaling pathway. This pathway regulates critical cellular processes, including proliferation, differentiation, apoptosis, and inflammation. Studies have shown that the MAPK pathway is activated in response to uremic toxins, leading to increased oxidative stress, endothelial injury, and inflammatory responses [35]. Specifically, activation of extracellular signal-regulated kinases (ERK1/2), c-Jun N-terminal kinases (JNK), and p38 MAPK have been implicated in vascular inflammation and endothelial dysfunction in CKD patients. Targeting components of the MAPK pathway has been proposed as a potential therapeutic strategy to mitigate the progression of cardiovascular diseases associated with CKD. Understanding the role of this pathway could provide valuable insights into the molecular mechanisms driving endothelial dysfunction and help identify novel targets for intervention. In this work, we hypothesized that by comparing the gene expression profile of human coronary artery endothelial cells (HCAECs) in RNA samples exposed to uremic serum obtained from CKD patients with and without MI, we could identify which genes are dysregulated and the signaling pathways that are likely to be associated in this in vitro model of endothelial dysfunction.

## 2. Results

### 2.1. Microarray Analysis, Overview

We obtained the genome-wide transcription of HCAECs that were exposed to 20% uremic serum for 48 h. RNA was isolated, transcribed into cDNA, and hybridized on an Affymetrix GeneChip^®^ Human Gene 1.0 ST array. A total of six microarrays were analyzed, identified as experiments 433, 434, and 435, corresponding to uremia without infarction (UWOI Group), and experiments 436, 437, and 438, corresponding to uremia with infarction (UWI Group). Microarray data have been deposited in the National Center for Biotechnology Information (NCBI) and are available in Gene Expression Omnibus (GEO), accession number GSE125898.

### 2.2. Quality Control Assurance

**Figure 1:**
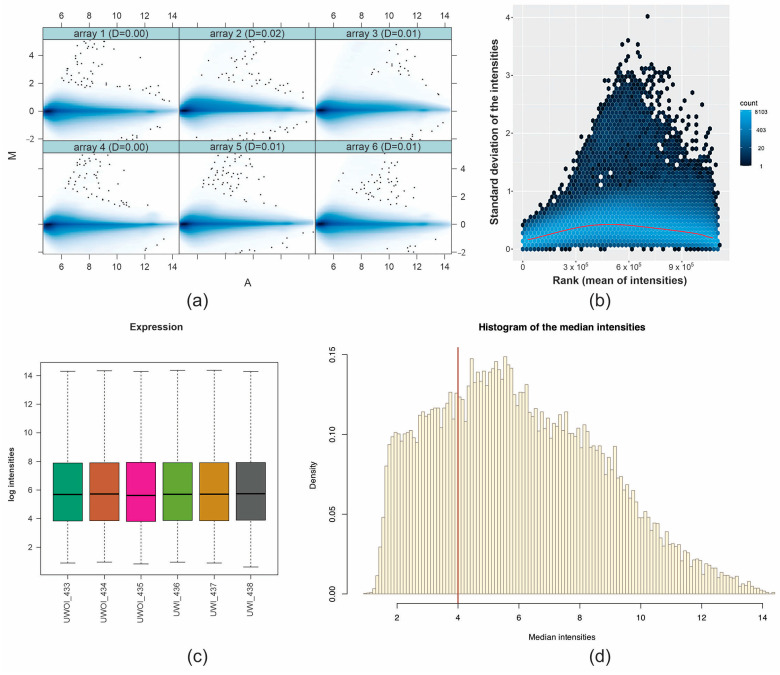
Graphical representation of quality control evaluation of microarrays (**a**) MA plots of six microarrays corresponding to the samples of the uremia without infarction (UWOI) and uremia with infarction (UWI) groups. Each dot represents a gene. The M-values are centered at zero, which, like (**b**), means that there is no dependence between the intensities and the logarithmic relationship. (**c**) Box plots of intensity level and probe density distribution among microarrays. (**d**) Density plot of the median intensities of the six microarrays.

Before determining differentially expressed genes (DEGs), we analyzed the microarray to assess its quality. Microarray quality analysis was performed with Bioconductor’s arrayQualityMetrics package. The quality was satisfactory, as can be seen in Figure 1.

### 2.3. Differential Gene Expression (DGE) Induced by Uremic Serum

**Figure 2:**
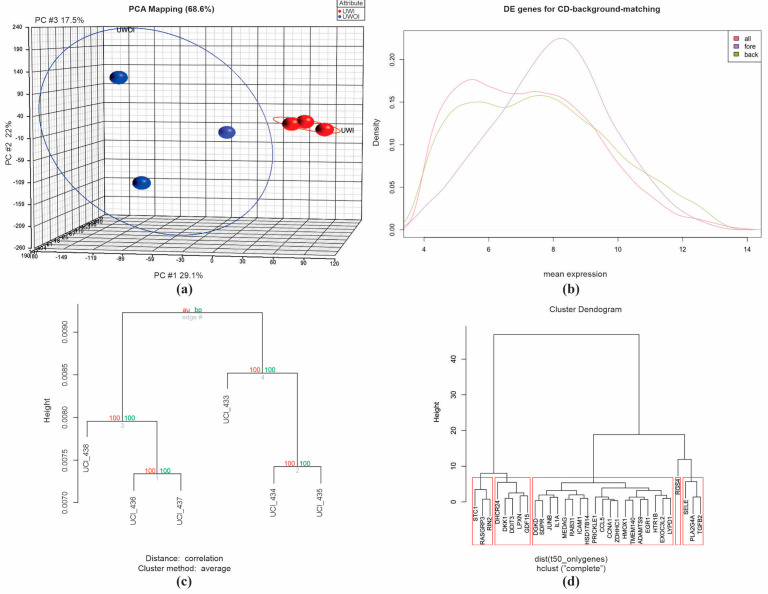
Principal component analysis (PCA) and comparison of gene expression profiles across hierarchical groups. (**a**) PCA describing the associated profile in the groups. In blue is the UWOI group, and in red is the UWI group. This panel (**a**) shows the difference in gene expression between the two study groups. Patients with uremia and infarction (UWI) showed more intergroup-related gene expression, unlike the uremia without infarction group (UWOI). (**b**) Plot of differentially expressed genes between density versus mean expression. All genes (red line), fore (purple line), and back (green line). (**c**,**d**) Dendrograms based on the expression of 100 and 50 genes, respectively. We used these genes as target genes for gene ontology and annotation analysis based on the expression of 50 genes (**d**) and 100 genes (**c**) involved in UWI visualized through a hierarchical clustering dendrogram of the expression profile.

Initially, in an unsupervised analysis, we identified 16,186 genes differentially expressed (DEGs) in HCAEC cells cultured in the presence of uremic serum from the two study groups (2.0-fold, *p* < 0.05). According to three independent statistical algorithms (*t*-test, Wilcoxon sum rank test, and distinction calculation) and the estimated FDR, we identified 100 genes involved in CKD disease through a principal component analysis (PCA) and a comparison of gene expression profiles across hierarchical groups. The cluster study using the PCA shows the grouping of the samples according to their differences: UWOI and UWI, which are located in opposite areas in the graph (Figure 2a). However, the UWOI group showed high intragroup dispersion that differed from that observed in the UWI group. Differences in the gene expression pattern could be related to abnormalities specific to each group (Figure 2b). We used two-way, unsupervised hierarchical clusters to analyze the expression profiles of the different groups. The unsupervised analysis clearly separates the two experimental groups, UWOI and UWI, demonstrating their dissimilarity. We used these genes as target genes for gene ontology and annotation analysis based on the expression of 50 genes (Figure 2d) and 100 genes (Figure 2c) involved in ESRD disease and infarction visualized through a hierarchical clustering dendrogram of the expression profile.

**Figure 3:**
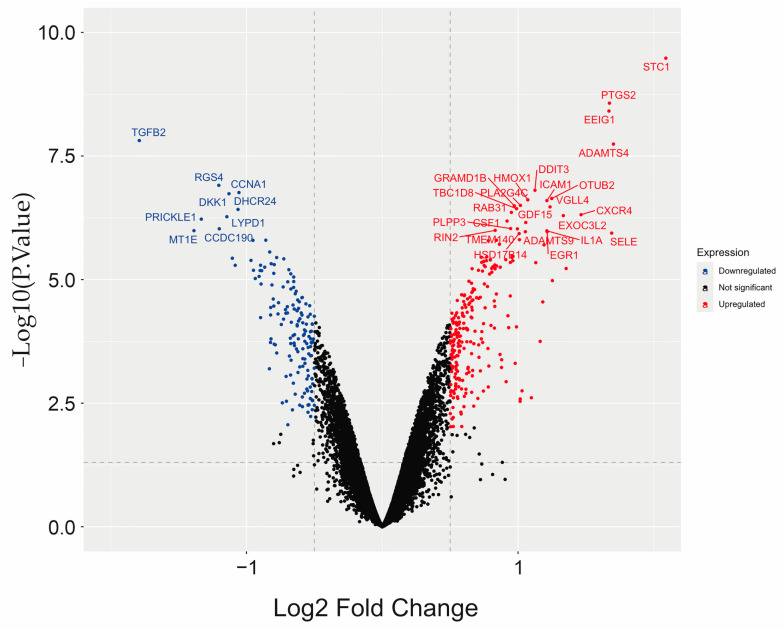
Volcano plot of genes that qualified as differentially expressed (DEGs) between UWOI and UWI groups in HCAECs. Blue dots in the upper left quadrant represent down-regulated genes, red dots in the upper right quadrant represent up-regulated genes, and black dots present stable genes (*p* < 0.05).

An analysis was then performed with the LIMMA package; in this case, a design of the experiment matrix was created, contrasting the UWI and UWOI cases. A linear model was developed for the data according to this contrast, and the empirical Bayes statistics for the differential expression test were applied. In this study, volcano plots were used to visualize the differentially expressed transcripts in the samples of the UWOI and UWI groups with the most significant data according to the adjusted *p*-value (with which the analysis of networks and ontology was carried out) (Figure 3).

**Figure 4:**
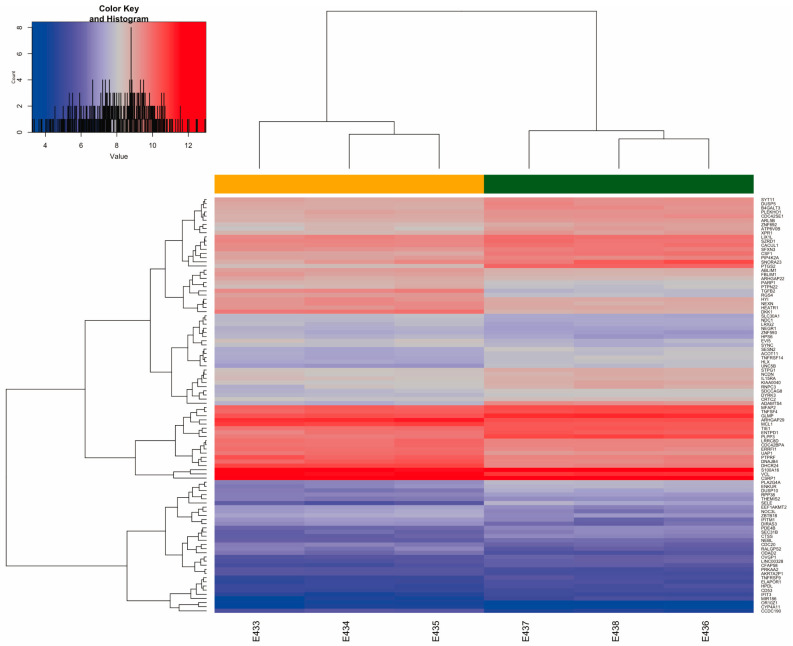
Hierarchical cluster. DEG heatmap of supervised analysis of 100 differentially expressed genes between UWOI (orange upper bar) and UWI (dark green upper bar) groups in HCAECs. The upper left quadrant shows the color key and histogram representing the behavior of DEGs for the UWOI and UWI groups in the HCAEC model. The samples are in the columns, and the genes are in the rows. Red color represent up regulated genes, and blue color represent down-regulated genes with different expression intensity.

Two-dimensional hierarchical clustering revealed a significantly different expression profile of 100 DEGs between the two groups of samples: 51 genes were overexpressed, and 49 were under-expressed in the UWI group. From these, the most significant upregulated coding genes were *STC1, ADAMTS4, SELE, PTGS2, CXCR4, UBD*, and *TLR2*; the most significantly downregulated genes were *TGFB2, MTIE, RGS4, LYPD1, DKK1*, and *SULTIB1*. Of the 100 most significant differentially expressed genes, it can be seen that the Staniocalcina-1 *(STC1,* stimulates renal phosphate reabsorption and could, therefore, prevent hypercalcemia, and his protein has been implicated in various biologic processes, including angiogenesis, bone and muscle development, and cellular metabolism) gene was the most overexpressed, with a logFC of 2.09, while the *TGFB2* gene (leading to recruitment and activation of SMAD family transcription factors that regulate gene expression, and including protein homodimerization activity and signaling receptor binding, the multifunctional protein regulates various processes such as angiogenesis and heart development) was the most under-expressed, with a logFC of −1.8 (Figure 4).

We consider significant genes in the framework of *p* < 0.05 and genes over-expressed with logFC > 2 and under-expressed with logFC < −2 to define differential gene expression profiles, as shown in Table 1.

**Table 1:** Top-ranked up-and-down-regulated differentially expressed genes identified by microarrays.

Symbol	Gene Name	LogFC *	Adj.*p*.Value	Regulation
STC1	stanniocalcin 1	2.0884	5.37 × 10^−6^	Up
ADAMTS4	ADAM metallopeptidase with thrombospondin type 1 motif 4	1.7026	5.87 × 10^−5^	Up
SELE	selectin E	1.6895	5.58 × 10^−4^	Up
PTGS2 ^b,i^	prostaglandin-endoperoxide synthase 2	1.6729	2.10 × 10^−5^	Up
EEIG1	estrogen-induced osteoclastogenesis regulator 1	1.6695	2.10 × 10^−5^	Up
CXCR4 ^j^	C-X-C motif chemokine receptor 4	1.4639	4.11 × 10^−4^	Up
TLR2 ^b^	toll-like receptor 2	1.2530	1.93 × 10^−3^	Up
OTUB2	OTU deubiquitinase, ubiquitin aldehyde binding 2	1.2478	3.41 × 10^−4^	Up
VGLL4	vestigial like family member 4	1.2355	3.63 × 10^−4^	Up
EGR1	early growth response 1	1.2146	5.44 × 10^−4^	Up
ICAM1 ^c,j^	intercellular adhesion molecule 1	1.2124	3.41 × 10^−4^	Up
IL1Ac ^j^	interleukin 1 alpha	1.2121	5.44 × 10^−4^	Up
CCL5 ^c^	C-C motif chemokine ligand 5	1.1912	7.83 × 10^−4^	Up
CYP1A1	cytochrome P450 family 1 subfamily A member 1	1.1822	3.63 × 10^−3^	Up
DDIT3	DNA damage-inducible transcript 3	1.1244	3.29 × 10^−4^	Up
MIR21 ^b,c,g,j^	microRNA 21	1.0981	5.19 × 10^−2^	Up
HMOX1 ^J^	heme oxygenase 1	1.0718	3.41 × 10^−4^	Up
GDF15	growth differentiation factor 15	1.0566	4.72 × 10^−4^	Up
ADAMTS9 ^j^	ADAM metallopeptidase with thrombospondin type 1 motif 9	1.0539	5.44 × 10^−4^	Up
PLA2G4C	phospholipase A2 group IVC	1.0174	3.63 × 10^−4^	Up
HSD17B14	hydroxysteroid 17-beta dehydrogenase 14	1.0079	5.58 × 10^−4^	Up
LIF ^j^	LIF interleukin 6 family cytokine	0.9787	2.07 × 10^−2^	Up
B3GAT3 ^e^	beta-1,3-glucuronyltransferase 3	0.9618	1.25 × 10^−3^	Up
MEF2Cd ^j^	myocyte enhancer factor 2C	0.9556	1.23 × 10^−3^	Up
CYP4A11	cytochrome P450 family 4 subfamily A member 11	0.9356	1.77 × 10^−3^	Up
VEGFA ^a,f,h,j^	vascular endothelial growth factor A	0.8306	1.32 × 10^−3^	Up
SEMA7A ^c,j^	semaphorin 7A (JohnMiltonHagen blood group)	0.8249	3.56 × 10^−3^	Up
TNFAIP3 ^j^	TNF alpha-induced protein 3	0.6544	5.62 × 10^−3^	Up
LPL ^b^	lipoprotein lipase	0.6376	3.97 × 10^−3^	Up
MAPK13 ^b^	mitogen-activated protein kinase 13	0.6326	2.29 × 10^−2^	Up
IFRD1 ^j^	interferon related developmental regulator 1	0.6210	2.71 × 10^−2^	Up
NEK10 ^a^	NIMA-related kinase 10	0.6161	0.04 × 10^−2^	Up
CEACAM1 ^g, j^	CEA cell adhesion molecule 1	0.5707	0.03 × 10^−2^	Up
TGFB1 ^a,b,c,d,e,g,j^	transforming growth factor beta 1	0.5671	1.11 × 10^−2^	Up
DUSP5	dual specificity phosphatase 5	0.5687	6.67 × 10^−3^	Up
ETS1 ^b,j^	ETS proto-oncogene 1, transcription factor	0.5425	6.76 × 10^−3^	Up
MMP14 ^d^	matrix metallopeptidase 14	0.5033	1.14 × 10^−2^	Up
PLAUR ^g^	plasminogen activator, urokinase receptor	0.5015	2.24 × 10^−2^	Up
CDK5 ^j^	cyclin-dependent kinase 5	0.4981	7.40 × 10^−3^	Up
ALPK3 ^f^	alpha kinase 3	0.4973	8.60 × 10^−3^	Up
TGFB2 ^f^	transforming growth factor beta 2	−1.7897	5.87 × 10^−5^	Down
MT1E	metallothionein 1E	−1.3871	5.44 × 10^−4^	Down
PRICKLE1 ^f,g,j^	prickle planar cell polarity protein 1	−1.3330	4.40 × 10^−4^	Down
RGS4 ^f^	regulator of G protein signaling 4	−1.2042	3.29 × 10^−4^	Down
CCDC190	coiled-coil domain containing 190	−1.2006	5.44 × 10^−4^	Down
LYPD1	LY6/PLAUR domain containing 1	−1.1449	4.12 × 10^−4^	Down
DKK1 ^a,i,j^	Dickkopf WNT signaling pathway inhibitor 1	−1.1293	3.29 × 10^−4^	Down
BDNF ^j^	brain-derived neurotrophic factor	−1.0834	1.30 × 10^−3^	Down
DHCR24	24-dehydrocholesterol reductase	−1.0626	3.63 × 10^−4^	Down
CCNA1	cyclin A1	−1.0558	3.29 × 10^−4^	Down
GPRC5A ^g^	G protein-coupled receptor class C group 5 member A	−0.8999	1.35 × 10^−3^	Down
CXADR ^f^	CXADR Ig-like cell adhesion molecule	−0.8661	1.30 × 10^−3^	Down
PDGFC ^a,c^	platelet-derived growth factor C	−0.8220	4.27 × 10^−3^	Down
OSMR ^b^	oncostatin M receptor	−0.8197	1.03 × 10^−2^	Down
PTGER4 ^b^	prostaglandin E receptor 4	−0.7993	1.47 × 10^−3^	Down
IGF1 ^c,d,f^	insulin-like growth factor 1	−0.7958	1.30 × 10^−3^	Down
TNIK ^j^	TRAF2 and NCK interacting kinase	−0.7303	2.35 × 10^−3^	Down
PHLDB2 ^j^	pleckstrin homology like domain family B member 2	−0.7131	4.56 × 10^−3^	Down
SEMA3C ^j^	semaphorin 3C	−0.6574	9.43 × 10^−3^	Down
FGD4 ^j^	FYVE, RhoGEF and PH domain containing 4	−0.6463	9.18 × 10^−3^	Down
FGF2 ^a,c,h,j^	fibroblast growth factor 2	−0.6167	4.35 × 10^−3^	Down
NRG1 ^c,d^	neuregulin 1	−0.6111	3.63 × 10^−3^	Down
PTPN22 ^c^	protein tyrosine phosphatase non-receptor type 22	−0.6083	2.28 × 10^−2^	Down
GLCE ^e^	glucuronic acid epimerase	−0.5920	3.28 × 10^−3^	Down
CCBE1 ^h,j^	collagen and calcium binding EGF domains 1	−0.5731	7.91 × 10^−3^	Down
BMP4 ^c,d,f,j^	bone morphogenetic protein 4	−0.5708	1.82 × 10^−2^	Down
IL1RL1 ^b^	interleukin 1 receptor like 1	−0.5655	8.30 × 10^−3^	Down
ARHGAP18 ^j^	Rho GTPase activating protein 18	−0.5625	3.88 × 10^−3^	Down
PRKDC ^j^	protein kinase, DNA-activated, catalytic subunit	−0.5411	6.17 × 10^−2^	Down
TBX18 ^f,j^	T-box transcription factor 18	−0.5347	4.10 × 10^−3^	Down
NTN4 ^j^	netrin 4	−0.5306	5.22 × 10^−3^	Down
SLIT2 ^j^	slit guidance ligand 2	−0.5214	1.64 × 10^−2^	Down
PRKCAc ^j^	protein kinase C alpha	−0.5177	3.13 × 10^−2^	Down
ABCC1 ^b^	ATP binding cassette subfamily C member 1 (ABCC1 blood group)	−0.5148	5.60 × 10^−2^	Down
LDLR ^b^	low-density lipoprotein receptor	−0.4993	2.04 × 10^−2^	Down
EFNB2 ^d,h,j^	ephrin B2	−0.4972	1.40 × 10^−2^	Down
ERBB2 ^a,g,j^	erb-b2 receptor tyrosine kinase 2	−0.4916	1.82 × 10^−2^	Down
MAP3K5 ^a^	mitogen-activated protein kinase kinase kinase 5	−0.4645	3.80 × 10^−2^	Down

* FC, fold change; ^a^ gene involved in positive regulation of *MAP* kinase activity; ^b^ gene involved in positive regulation of inflammatory response; ^c^ gene involved in positive regulation of ERK1 and ERK2 cascade; ^d^ gene involved in positive regulation of striated muscle cell differentiation; ^e^ gene involved in glycosaminoglycan biosynthetic process; ^f^ gene involved in cardiac muscle cell development; ^g^ gene involved in epidermal growth factor receptor signaling pathway; ^h^ gene involved in lymph vessel development; ^i^ gene involved in Regulation of synaptic transmission glutamatergic; ^j^ gene involved in regulation of anatomical structure morphogenesis (see Table 2).

**Table 2:** The top 10 best enriched biological processes.

GO ID	Term Description	Annotated	Significant	Rank in Fisher Classic	Fisher Elimination	Fisher Classic	Significant Genes
GO:0043406	Positive regulation of MAP kinase activity	27	15	86	0.00016	0.00016	*DKK1*; *ERP29*; *FLT1*; *ERBB2*; *TGFB1*; *IL1B*; *CD40*; *DVL3*; *NEK10*; *FGF2*; *PDGFC*; *GHR*; *EDN1*; *VEGFA*; *MAP3K5.*
GO:0050729	Positive regulation of inflammatory response	45	21	101	0.00023	0.00023	*TNFSF18*; *TNFSF4*; *PTGS2*; *ETS1*; *NFBIA*; *ABCC1*; *GPRC5B*; *NUPR1*; *ADOR2*; *MIR21*; *LDLR*; *TGFB1*; *IL1RL1*; *IL1B*; *TLR2*; *OSMR*; *PTGER4*; *NAIP*; *TNIP1*; *MAPK13*; *LPL.*
GO:0070374	Positive regulation of ERK1 and ERK2 cascade	61	26	107	0.00027	0.00027	*PTPN22*; *CAVIN3*; *DENND2B*; *IGF1*; *FERMT2*; *BMP4*; *SEMA7A*; *CCL2*; *MIR21*; *PRKCA*; *CCL5*; *ABCA7*; *ICAM1*; *MIR23A*; *TGFB1*; *IL1A*; *FGF2*; *RAPGEF2*; *PDGFC*; *TPBG*; *CCN2*; *GPNMB*; *MTURN*; *INHBA*; *NRG1*; *ABL1*.
GO:0051155	Positive regulation of striated muscle cell differentiation	15	10	110	0.00028	0.00028	*IGF1*; *EFNB2*; *MMP14*; *BMP4*; *TGFB1*; *TBX1*; *MEF2C*; *EDN1*; *NRG1*; *NIBAN2*.
GO:0006024	Glycosaminoglycan biosynthetic process	20	12	112	0.00028	0.00028	*CHST3*; *ST3GAL4*; *CHST1*; *B3GAT3*; *CHST11*; *GLCE*; *CLTC*; *TGFB1*; *IL1B*; *ST3GAL6*; *ABCC5*; *CHSY3*.
GO:0055013	Cardiac muscle cell development	37	18	120	0.00034	0.00034	*RGS4*; *NEBL*; *PRICKLE1*; *IGF1*; *BMP4*; *ALPK3*; *MEF2A*; *FHOD3*; *MIR23A*; *FHL2*; *CXADR*; *PDLIM5*; *SORBS2*; *EDN1*; *VEGFA*; *TBX18*; *BVES*; *PLEC*.
GO:0007173	Epidermal growth factor receptor signaling pathway	44	20	137	0.0005	0.0005	*ERRFI1*; *PIK3C2A*; *GPRC5A*; *PRICKLE1*; *BCAR1*; *ERBB2*; *MIR21*; *RHBDF2*; *MVB12A*; *TGFB1*; *CEACAM1*; *PLAUR*; *DGKD*; *CCDC88A*; *TGFA*; *CBLB*; *AREG*; *GAB1*; *ERBIN*; *ABL1*.
GO:0001945	Lymph vessel development	16	10	147	0.0006	0.0006	*TIE1*; *PTPN14*; *EFNB2*; *CLEC14A*; *TMEM204*; *CCBE1*; *TBX1*; *HEG1*; *FGF2*; *VEGFA*.
GO:0051966	Regulation of synaptic transmission glutamatergic	24	13	150	0.00062	0.00062	*PTGS2*; *DKK1*; *DGKZ*; *SYT1*; *CLN3*; *CCL2*; *CACNG7*; *SHANK3*; *HOMER1*; *MEF2C*; *HTR1B*; *CDK5*; *OPHN1*.
GO:0022603	Regulation of anatomical structure morphogenesis	352	103	175	0.00084	0.00084	*FBLIM1*; *TIE1*; *CSF1*; *STRIP1*; *PLEKHO1*; *TGFB2*; *LRP8*; *CDC42SE1*; *AKT3*; *DKK1*; *ADAM12*; *BDNF*; *ETS1*; *FGD4*; *SYT1*; *EPS8*; *PRICKLE1*; *BTG1*; *NTN4*; *FLT1*; *EFNB2*; *DAAM1*; *INF2*; *FERMT2*; *BMP4*; *PGF*; *SEMA6D*; *SEMA7A*; *EEF2K*; *CCL2*; *ERBB2*; *FMNL1*; *MIR21*; *PRKCA*; *RAC3*; *DLG4*; *SP6*; *SPAG9*; *DCC*; *NEDD4L*; *EPB41L3*; *CCBE1*; *EMC10*; *CACNG7*; *TGFB1*; *CEACAM1*; *CDC42EP3*; *IL1A*; *IL1B*; *CXCR4*; *STAT1*; *CD40*; *ADAMTS1*; *RUNX1*; *TBX1*; *NF2*; *HMOX1*; *SHANK3*; *LIF*; *PHLDB2*; *SKIL*; *DVL3*; *ADAMTS9*; *TNIK*; *SLIT2*; *PDLIM5*; *FGF2*; *GAB1*; *POU4F2*; *RAPGEF2*; *RASA1*; *MYO10*; *MEF2C*; *EFNA5*; *EDN1*; *VEGFA*; *TNFAIP3*; *TBX18*; *BVES*; *ARHGAP18*; *SYNE1*; *EZR*; *LFNG*; *ITGB8*; *GPNMB*; *IFRD1*; *NOS3*; *SEMA3C*; *SEMA3D*; *SMURF1*; *PLXNA4*; *CDK5*; *ADGRA2*; *SDC2*; *NEFL*; *PRKDC*; *PALM2AKAP2*; *ABL1*; *NIBAN2*; *NSMF*; *FOXO4*; *SH3KBP1*; *ZMYM3*.

### 2.4. Functional Analysis Through Gene Enrichment

To explore the functional similarities of the 100 DEGs, enrichment analysis on gene sets was performed using Gene Ontology (GO). The significance of the enrichment was measured by the *p*-value according to Fisher’s exact test. For 51 overexpressed genes in the UWI group, we identified 500 GO terms and a KEGG pathway with *p* < 0.05. The GO terms identified include positive regulation of MAP kinase activity (with 15 significant genes) and positive regulation of ERK1 and ERK2 cascade, regulation of cardiac muscle cell development (with 37 significant genes), Epidermal growth factor receptor signaling pathway, Regulation of anatomical structure morphogenesis (with 352 significant genes), among others (Table 2).

**Figure 5:**
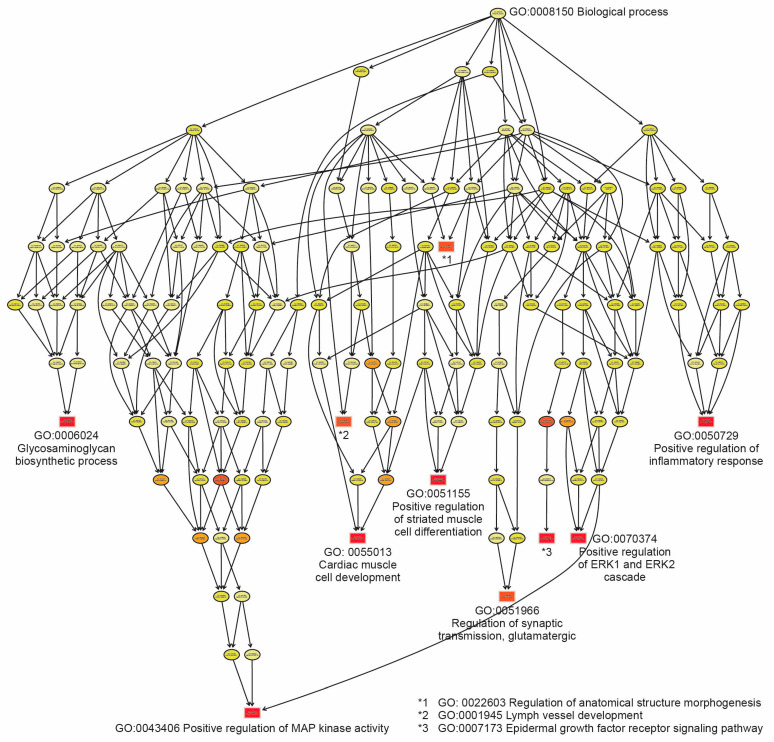
A map of the best enriched GO hierarchies according to the criterion of the classic Fisher test, defining the ten most significant enriched DEG term nodes (represented in red squares). In this figure, some categories are very general and do not mention the genes they include, but it can be seen that the MAPK category is the most enriched. The interpretation of Table 2 is greatly complemented by Figure 5.

Further analysis showed that the metabolic pathway most significantly represented (*p* = 0.00016) by Fisher’s test was the mitogen-activated protein kinase (MAPK) pathway (Figure 5).

### 2.5. Construction of a Network of Molecular Interactions

**Figure 6:**
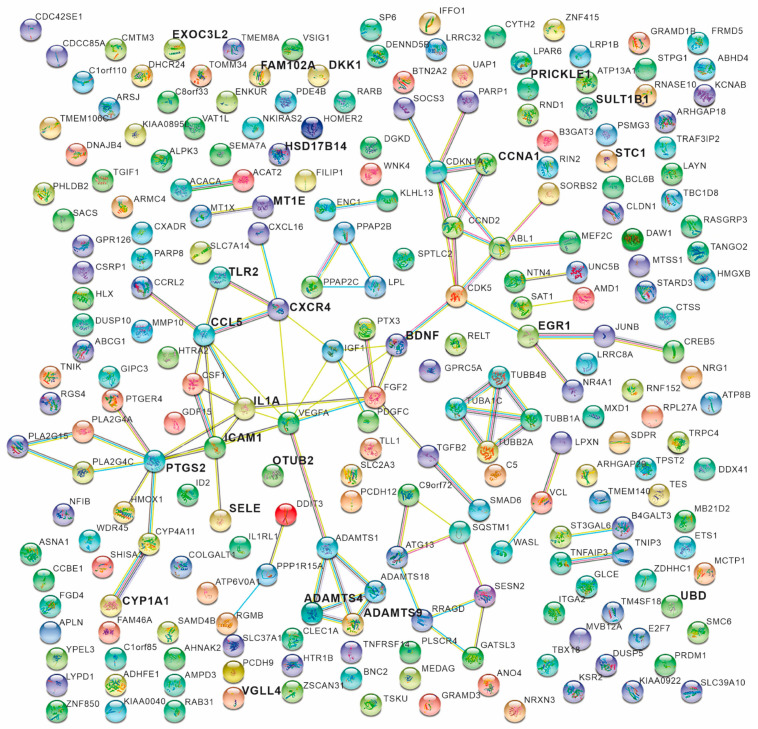
Molecular networks of HCAECs exposed to uremic serum. Associated genes derived from String were based on correlations in order to characterize cellular and molecular functions and identify enriched canonical pathways/networks for the list of selected candidate genes, according to Gen Ontology data. The solid lines represent the interactions between genes, and the nodes (spheres) represent the proteins that are associated with the respective genes. Each color of the nodes represents evidence of protein–protein interaction. Pink indicates experimentally determined/post-translational modifications; blue indicates gene co-occurrence; green indicates gene neighborhood; black indicates co-expression; red indicates gene fusion. Nodes with ribbon-like structures represent the availability of 3D structural information of the protein being predicted.

Molecular networks were algorithmically generated by the STRING platform (STRING; https://string-db.org/cgi/input.pl (accessed on 5 April 2025)) using significant GO terms and pathway analysis as instructive tools to comprehensively explore the molecular mechanisms involved in our study. As a result, some positively regulated genes and protein-encoded genes were obtained in the transduction network. Five genes—*CCL5*, *IL1A*, *PTGS2*, *BDNF*, *ICAM1*, and *ADAMTS4*—were observed among others as core genes, which are related to inflammation and mitogenesis, regulation of the stress response, cell adhesion, degradation of proteoglycan of cartilage, signal transduction, and transcriptional regulation in HCAEC cells stimulated with uremic serum (Figure 6).

### 2.6. Validation of DEGs Through Quantitative RT-qPCR

Based on the analysis of the uremic serum pathway and molecular networks in HCAECs, we randomly selected four candidate genes—*PTGS2*, *DDIT3*, *PLA2G4C*, and *EEIG1*—for the validation of gene expression through RT-qPCR analysis. We prioritize the validation of these genes according to the following characteristics: STC1 was prioritized by the magnitude of its fold change (FC) obtained (the gene was the most overexpressed, with a logFC of 2.09). Also, despite its overexpression in the molecular networks of HCAECs exposed to uremic serum that we performed with STRING, this gene is observed to be isolated, probably with a function not yet defined in this in vitro model. In addition, it has been implicated in various biological processes, such as angiogenesis, bone and muscle development, and cellular metabolism. In addition, in animal models, it produces elevated serum phosphate levels and an increased metabolic rate. *DDIT3*: also prioritized by its logFC = 1.124, and because it is a multifunctional transcription factor that plays an essential role in the response to a wide variety of cell stresses and induces cell cycle arrest and apoptosis in response to endoplasmic reticulum stress. Additionally, play a regulatory role in the inflammatory response through the induction of caspase-11 (CASP4/CASP11), which induces the activation of caspase-1 (CASP1), and both these caspases increase the activation of pro-IL1B to mature IL1B which is involved in the inflammatory response. In addition, it also participates as a major regulator of postnatal neovascularization through the regulation of endothelial nitric oxide synthase (NOS3)-related signaling. *EEIG1*: also known as *FAM102A*, it is also overexpressed in the uremia group with infarction (logFC = 1.6695) and is involved in the positive regulation of osteoclast differentiation. The key component of TNFSF11/RANKL- and TNF-induced osteoclastogenesis pathways thereby mediate bone resorption in pathological bone loss conditions. Also, despite its overexpression in the molecular networks of HCAECs exposed to uremic serum that we performed with STRING, this gene is observed as isolated, probably with a function not yet defined in this in vitro model. *PLA2G4C*: this gene was prioritized because it has a fold change of 1.017 and is involved in phospholipid remodeling with implications in endoplasmic reticulum membrane homeostasis and lipid droplet biogenesis to produce free fatty acids and lysophospholipids, both of which serve as precursors in the production of signaling molecules and are predominantly expressed in cardiac and skeletal muscle. In the Molecular Networks of HCAECs exposed to the uremic serum that we performed with STRING, this gene is observed to be associated with the central node of PTGS2.

The results showed that the expressions of the four genes increased in the UWI group and were underexpressed in the UWOI group in HCAEC cells treated with uremic serum. These results were in line with those obtained through microarray analysis.

## 3. Discussion

Cardiovascular disease is a major cause of death in ESRD patients. It is widely recognized that patients on dialysis have substantially higher cardiovascular and non-cardiovascular mortality rates compared with the general population, accounting for approximately 50% of total mortality [24,25,26], and little is known about the molecular mechanism involved in these events. In this study, we used a microarray approach to identify HCAEC gene expression signatures after the exposition of serum samples with and without MI. We also identified the MAPK signaling pathway as the most enriched pathway in this study, with statistical significance, and found that it may be linked to genes involved in important physiological processes in ESRD and CVD. According to the GO classification, we found that genes differentially express a variety of transcription factors that are involved in the immune response. Several studies have discussed the role of inflammation as a first step in promoting endothelial dysfunction and the progression of atherosclerotic processes. Additionally, some studies suggest that atherosclerosis could be caused by an immune reaction against autoantigens such as oxidized low-density lipoproteins and heat shock proteins [39].

Interestingly, our microarray profile highlights several genes that could sustain common molecular alterations in ESRD and CVD, some of which were validated by RT-qPCR analysis. The cardiovascular system is the main target of uremic toxins and chronic inflammation in ESRD patients. To our knowledge, genetic studies focused on endothelial dysfunction associated with cardiovascular development in ESRD patients are scarce. One notable study is that reported by Cardinal et al., the results of which suggest a possible mechanism involved in patients with ESRD and endothelial dysfunction, apparently through six genes associated with the regulation of cell cycle progression (CDK-1, topoisomerase II, PDZ-binding kinase, CDCA1, protein SDP35, and transcription factor E2F) and two cholesterol exit system genes (ABCA1 and ABCG1), which are deregulated in HCAECs exposed to uremic plasma [36]. Other studies have shown that permanent Endothelial cell aggression as a result of chronic exposure to uremic toxins induces cellular phenotype abnormalities, which may result in high serum levels of inflammatory biomarkers such as IL-8 and MCP-1, cytokines, and the adhesion molecules VCAM-1 and ICAM-1 [40]. The main finding of this work was the identification of key genes involved in the activation of the MAPK signaling pathway. The MAPK signaling pathway is involved in a repertoire of biological events, including proliferation, differentiation, metabolism, motility, survival, and apoptosis. This pathway encompasses a large number of serine/threonine kinases and is divided into four MAPK subfamilies, including ERK1/2, c-Jun NH2-terminal kinases (JNK1, -2, and -3), p38 kinase (α, β, γ, and δ) and big MAPK (BMK or ERK5) [41]. Studies have shown that MAPK subfamilies are involved in the pathogenesis of numerous renal diseases, including CKD and ESRD [42], and produce important signaling molecules involved in the inflammatory process in the kidney. Likewise, previous studies have focused on TGF-β and epithelial or endothelial cells for mesenchymal transition in myofibroblast transformation, which leads to fibrosis [43]. One important gene found among the 15 significant genes of the MAP pathway was MAP3K5 (Serine/threonine kinase, which acts as an essential component of the MAP kinase signal transduction pathway). Plays a crucial role in the apoptosis signal transduction pathway through mitochondria-dependent caspase activation and mediates signal transduction of various stressors like oxidative stress as well as by receptor-mediated inflammatory signals. Moreover, together with ASK1, it is a serine/threonine kinase, MAP3K family member, which induces apoptosis through the activation of JNK and p38. ASK1 has been implicated in the pathology of neurodegenerative and oxidative stress-related diseases [43].

On the other hand, while searching for a set of genes associated with the MAPK signaling pathway, we found a group of four DEG members of this pathway: PLA2G4A, IL1A, RASGRP3, and DDIT3, which are molecules related to inflammation, apoptosis, signal transduction, and atherosclerosis. PLA2G4A was one of the two most significantly overexpressed genes. Phospholipases A2 (PLA2s), a family of enzymes that hydrolyze the fatty acid at the sn-2 position of phospholipids, play pivotal roles in cell signaling and inflammation [44]. Recently, these enzymes have also been reported to function as key regulators of lipid droplet homeostasis [45]. Although various cellular PLA2s may contribute to the generation of free fatty acids from membrane phospholipids initially required for lipid droplet synthesis, there is strong evidence to support that the PLA2 form, such as PLA2G4A, is also involved in endothelial reticulum phospholipid remodeling and lipid droplet expansion processes [41,43]. IL1A was also significantly overexpressed in this analysis. This gene encodes for Interleukin-1 (IL-1), a proinflammatory cytokine that plays a crucial role in ischemic stroke [46]. Given that intracranial atherosclerosis is a risk factor for ischemic stroke [47], this finding strongly suggests that IL-1 is implicated in the pathophysiology of ischemic stroke. In our study, the guanylyl-nucleotide-releasing protein RAS-3 (RASGRP3) was one of the main overexpressed genes associated with the MAPK pathway. Members of the RAS subfamily of GTPases function as signal transducers, as GTP/GDP-regulated switches that cycle between GDP-bound inactive states and GTP-bound active states, serve as activators of RAS by promoting GTP acquisition to maintain the GTP-bound active state, and are the key link between cell surface receptors and RAS activation [46]. DNA-damage-inducible transcript 3 (DDIT3) was also significantly overexpressed in this analysis. This gene encodes a member of the CCAAT/enhancer-binding protein (C/EBP) family of transcription factors. The protein functions as a dominant-negative inhibitor by forming heterodimers with other C/EBP members, such as C/EBP and LAP (liver activator protein), and preventing their DNA-binding activity. During endoplasmic reticulum stress (such as in pancreatic beta cells or in atherosclerosis-associated macrophages), CHOP can induce Ero1 activation, leading to calcium release from the endoplasmic reticulum into the cytoplasm, resulting in the activation of apoptosis [47,48]. A study showed a significant increase in the expression of the ddit3 protein, which is a transcription factor that could regulate a number of inflammatory cytokines, such as IL-6 [33]. Interestingly, the biological network generated by the String software platform showed an important functional role in all processes described above. Finally, our results revealed a certain genetic profile with a small set of genes that, in the future, could provide additional information on the biological basis of CVD in CKD.

### Limitations of the Study

We consider the main limitation of this study to be the small number of samples and microarrays analyzed. However, the in vitro studies were performed with technical replicates to minimize variations and were confirmed using different statistical approaches. It is worth noting that our small sample size is characteristic of a comparative, non-predictive experimental study conducted in an in vitro model. We believe this sample size is adequate for exploring the genes involved in the uremia condition, both with and without myocardial infarction, given the type of microarray used in our research. We have undertaken a formal analysis of the sample’s power, aimed at identifying some potential biomarkers for myocardial infarction in patients with ESRD. This allows us to attribute the differences in gene expression mainly to infarction. This design is aligned with the goal of understanding the effects of infarction within the context of ESRD. We acknowledge our results cannot be applied to patients without ESRD. Although the sample size per group is small, the inclusion of individuals of both genders reduces bias due to possible gender-related differences in expression. This approach also increases the likelihood of reproducibility by keeping the demographic composition constant.

Another limitation of this study is that we did not include serum samples from patients without ESRD. We know that in the design of this comparative in vitro experimental study, serum samples from two groups of patients on KDIGO stage G4–G5 in renal replacement therapy treatment were compared: (1) uremic group with infarction: patients with advanced chronic kidney disease and history of infarction and (2) Uremic group without infarction (control): Patients with ESRD but without a history of infarction. The uremic group without infarction was used as a control to evaluate changes in gene expression attributable to infarction in the context of uremia. The rationale for including this control group is mainly due to two situations: (1) For the similarity of the clinical conditions of the two groups, since we consider that the choice of patients with uremia stage KDIGO G4–G5 as a control group is appropriate because they share metabolic and inflammation alterations that are characteristic of ESRD, which allows us to attribute the differences in gene expression mainly due to infarction, and (2) For comparative relevance, as this design is aligned with the goal of understanding the effects of infarction within the context of ESRD. Moreover, the nature and spectrum of cardiovascular disease in CKD are recognized to be different from that in people without kidney disease, including atherosclerosis, arteriosclerosis, calcific arterial and valve disease, left ventricular remodeling and dysfunction, arrhythmia, and sudden cardiac death.

We acknowledge that part of the limitations of choosing this Control Group may be mainly due to: (1) reduced biological variability (although while confounders can be minimized, uremia-specific gene alterations could mask differences related exclusively to infarction), and (2) generalizability (the results cannot be applied to patients without ESRD). It should be noted that additional recommendations for further (non-experimental) studies should be: (1) Include a healthy reference group: Incorporating an additional group of patients without renal disease could provide a benchmark for understanding how uremia and infarction interact. (2) Confirmation of the results: Validate the findings by quantitative PCR or other techniques in a larger sample size. While the inclusion of a healthy control group could have added value to our study, our primary focus is on understanding why certain patients with the disease experience infarction. Consequently, comparing uremic patients who do not have infarction with those who do allows for a more straightforward investigation of the biological processes involved, minimizing variation and enhancing our understanding of the gene expression changes induced by uremic serum, which is present in both patient groups. In many diseases, sourcing samples from healthy individuals without the disease of interest can pose challenges. Although this may not be a significant concern in our study, it can introduce additional variability in the “healthy” samples that may not be helpful in precisely identifying the genes implicated in the development of infarction. Nonetheless, these samples could provide valuable insights into the genes affected by uremic toxins.

Another limitation of the study is that plasma levels of inflammatory markers such as IL-6, TNF-alpha, and C-reactive protein, among others, were not determined. However, the role of these cytokines in the pathophysiological mechanisms of cardiovascular disease in patients with ESRD is known [49,50].

## 4. Materials and Methods

### 4.1. Origin and Collection of Human Uremic Serum

After an initial screening, only six patients undergoing conservative renal replacement therapy (RRT) for chronic kidney disease (CKD) at the dialysis outpatient clinic of the UMAE-Specialty Hospital, the National Medical Center Siglo XXI (CMNS-XXI), and the Mexican Social Security Institute (IMSS), Mexico City, were included in this study. They were divided into two groups: Group 1 (“uremia without infarction”, UWOI): Three patients with ESRD on continuous ambulatory peritoneal dialysis (CAPD) with no history of myocardial infarction (MI). MI was ruled out based on clinical signs, color Doppler echocardiography, and biochemical parameters. Group 2 (“uremia with infarction”, UWI): Three patients with ESRD undergoing hemodialysis (three sessions per week) who had experienced an MI within the past year. Infarction was confirmed through clinical signs, electrocardiography, color Doppler echocardiography, and biochemical parameters. None had received thrombolysis due to late hospital arrival, and at the time of the study, none exhibited precordial pain. All had been diagnosed with anterolateral wall MI. All subjects had normal kidney function and no history of vascular disease, diabetes, dyslipidemia, or smoking. They used no medication. Inclusion criteria were: age ≥ 18 years, CKD diagnosis (defined by proteinuria or persistently decreased glomerular filtration rate [GFR] in three consecutive evaluations), and informed consent; exclusion criteria were the presence of active inflammatory/infectious disease (determined by the absence of clinical signs of acute inflammation), malignant neoplasia, immunosuppressive drug use, or other conditions, and elimination criteria were: refusal to participate in the study. One patient in group 1 whose medical record had a diagnosis of ischemic heart disease could not be confirmed at the time of inclusion in the study. GFR was calculated as the average of two measurements of mean urea and creatinine clearance. Primary renal disease was diagnosed through a thorough clinical assessment.

### 4.2. Sample Collection and Processing

Management, collection and provenance of human serum blood samples were collected from volunteers and ESRD patients on thrice-weekly chronic hemodialysis or CAPD. All participants provided informed consent. Blood was collected in 10 mL tubes dry, sent on ice to the laboratory, and centrifuged (1300× *g* for 10 min). The serum was then aliquoted and stored at −80 °C. The serum was not pooled. Each serum was used for an individual experiment. Two different subsets of ESRD patients provided serum for the experiments conducted in this study. The characteristics of patients providing serum in the two uremic groups are provided in Table 3. Their mean age was 40.0 ± 5.0 years, and 66% were of female gender. They were matched for gender and age (5-year caliper) with each uremic subject. For the ribonucleic acid (RNA) extraction and measurement of biochemical parameters, a 10 mL venous blood sample was drawn from the antecubital vein of the left arm using a 10 mL syringe and collected in tubes without anticoagulant (BD-Vacutainer, Plymouth, UK). Samples were transported on ice to the laboratory and centrifuged at 1300× *g* at 4 °C for 10 min to obtain serum, which was aliquoted and stored at −80 °C until further use.

The study protocol was approved by the Research Ethics Committee of CMNS-XXI, IMSS (Approval Number: R-2008-3601-113/FIS/IMSS/PROT/551). All participants provided written informed consent before enrollment. Initially, for the design, we have undertaken a formal analysis of the sample’s power. Due to that, this is a comparative, non-predictive experimental study aimed at identifying a potential biomarker for acute myocardial infarction in patients with end-stage renal disease (ESRD). Following our theoretical analysis, we created a notebook with R code and provided justifications through the analysis of microarray data. Sample size estimation methods and ideas were taken from Lin, W.J., et al. [51]. This process closely resembled the one proposed in this portal: https://bioinformatics.mdanderson.org/MicroarraySampleSize/ (accessed on 8 February 2021), with the selected options reflecting those outlined in this analysis [52,53].

Recognizing that the primary objective of this study is to establish an initial framework for identifying relevant genes rather than offering a highly discriminatory and statistically significant description, we opted to reduce the sensitivity value. Our intention is to include a broader range of genes for further examination.

It is worth noting that our small sample size was characteristic of a pilot study conducted in an in vitro model. We believe this sample size was adequate for exploring the genes involved in the uremia condition, both with and without infarction, given the type of microarray used in our research. To ensure statistical power, for the design, we used the online software from the M.D. Anderson Bioinformatics with the data as follows: number of genes, 28,869; acceptable number of false positive, 10%; desired fold differences, 2; desired power, 0.7; standard deviation of 0.5, which compute a sample size per group of 3 with a per-gene value alpha of 0.09997.

The characteristics of the selected patients who provided a peripheral blood sample in the two uremic groups are shown in Table 3.

### 4.3. Primary Culture of Human Endothelial Cells, RNA Isolation, Purification and Quality

Human coronary arterial endothelial cells (HCAECs) (PCS-100-020) and Endothelial Cell Enrichment Kit-VEGF medium (PCS-100-041) were purchased from the American Type Culture Collection (ATCC, Manassas, VA, USA). HCAEC culture was performed according to the manufacturer’s instructions. Briefly, HCAECs proliferated in the enrichment medium with final medium concentrations as follows: basic rhFGF, 5 ng/mL; rhIGF-1, 15 ng/mL; L-glutamine, 10 mM; heparin sulfate, 0.75 U/mL; hydrocortisone hemisuccinate, 1 µg/mL; fetal bovine serum, 2%; ascorbic acid, 50 µg/mL; penicillin–streptomycin, 10 U/m-10 µg/mL. Cell cultures were maintained in a humidified atmosphere containing 5% (*v*/*v*) CO_2_ incubator at 37 °C, and the culture medium was renewed every 2 days, with a 1:3 split at each passage, and only cells from 3 to 6 passages were used. When monolayers were 80 to 90% confluence in culture plates, they were exposed to DMSO (as sample internal control). After, the serum sample obtained from each patient included in the UWOI and UWI groups was inactivated for 30 min at 56 °C, adjusted to a concentration of 20% with endothelial enrichment-VEGF medium, and then exposed to cell cultures and incubated for 48 h. To exclude that serum exerted direct toxic effects on HCAECs, trypan blue exclusion assays [54] were performed at the conclusion of the incubation and demonstrated > 95% viability with no differences between our experimental groups. At the end of exposure, the cells and supernatants were collected and stored at −80 °C until use. After exposure, total RNA was isolated using a RNeasy kit, Qiagen (QIAGEN, Valencia, CA, USA), following the manufacturer’s protocol and stored at −80 °C until use. The RNAs used in this study were selected and referred to as Experiments 433, 434, and 435, corresponding to the UWOI group, and Experiments 436, 437, and 438, corresponding to the UWI group, respectively. First, the chemical purity of the RNA quality was checked with a Nanodrop ND-1000 spectrophotometer at 260 nm (NanoDrop Technologies, Wilmington, DE, USA). RNA integrity was assessed with RNA integrity number (RIN) scoring using an Agilent 2100 Bioanalyzer, RNA 6000 Nano LabChip kit, and Agilent 2100 Expert Software (Agilent Technologies, Santa Clara, CA, USA). Only samples with RIN ≥ 9.5 were included in the microarray assay.

### 4.4. Microarray Assay

The microarray used for our analysis was Affymetrix GeneChip^®^ Human Gene 1.0 ST array (Affymetrix, Santa Clara, CA, USA), which interrogates 28,869 well-annotated genes with 764,885 distinct probes. RNA sample transcription, microarray hybridization, washing, staining and scanning of microarrays were performed according to the manufacturer’s guidelines (Affymetrix, GeneChip^®^ Whole-Transcript (WT) Sense Target Labeling Assay User Manual, P/N 701,880 Rev.5). Briefly, 100 ng of total RNA was amplified and labeled using the Affymetrix GeneChip^®^ WT Terminal Labeling Kit (P/N 900671). Then, the final labeled target DNA was hybridized to the arrays in a GeneChip^®^ hybridization oven 640 (Affymetrix, Santa Clara, CA, USA) at 60 rpm and 45 °C for 17 h. Subsequently, the microarrays were washed and stained using the GeneChip^®^ Fluidics Station 450 (Affymetrix, Santa Clara, CA, USA) and scanned using a GeneChip^®^ Scanner 3000 7 G (Affymetrix, Santa Clara, CA, USA). A total of 6 microarrays were analyzed. The named experiments 433, 434, and 435 correspond to the ESRD-UWOI Group, and Experiments 436, 437, and 438 correspond to the ESRD-UWI Group. GeneChip^®^ (http://www.affymetrix.com/support/technical/datasheets/human_datasheet.pdf (accessed on 5 April 2025)) operating software (GCOS, Affymetrix) was used to obtain and analyze the images. Scanning and data extraction of the microarray were followed by the transformation of fluorescence data into CEL files (probe CEL intensity data) employing Affymetrix GeneChip^®^ Command Console (AGCC) software (Affymetrix Inc. Santa Clara, CA, USA).

### 4.5. Data Preprocessing

The raw data from the CEL files were converted into expression values, background corrected, and quantile normalized through a custom pipeline implemented using the Affyparser, Affyoi, and Oligo packages of R/Bioconductor [55]. The robust multiarray average (RMA) algorithm was implemented through the execution of background correction and subsequent filtering to remove transcripts with very low expression levels, followed by quantile normalization and finished with probe summarization, for which a cutoff value of 4.2 was used for the median of intensities. Subsequently, annotation of the transcripts was carried out using the annotation data of Affymetrix hugene10sttranscriptcluster.db (R package version 8.7.0.).

### 4.6. Differentially Expressed Genes (DEGs) in UWOI and UWI Groups

A linear model was implemented to evaluate statistically significant changes in the expression of genes between ESRD groups. The LIMMA (Linear Models for Microarray Data) package of R/Bioconductor [56] was performed to assess the differential gene expression (DGE) values between uremia without infarction (UWOI) and uremia with infarction (UWI) using the Bayesian empirical method. First of all, a contrast matrix was generated between the UWOI and UWI groups, and then model fitting was performed. Statistical relevance was determined with the Bayes variance moderation method using a moderate Student *t*-test (which is recommended for variance estimation in microarray experiments with few replicates). A false discovery rate (FDR) > 0.1 was taken into account as a criterion to obtain only significant adjusted *p*-values. The threshold of DEGs was set according to a log_2_FC (fold change) value > 1, a log_2_FC value < −1, and a *p*-value ≤ 0.01. Volcano plots were used to visualize the analysis.

### 4.7. Pathway Enrichment Analysis of DEGs

The screened DEGs were loaded into the Database for Annotation, Visualization, and Integrated Discovery (DAVID) (available at https://davidbioinformatics.nih.gov (accessed on 4 May 2021)) for Gene Ontology (GO) annotation and pathway enrichment analysis from the Kyoto Encyclopedia of Genes and Genomes database (KEGG, http://www.genome.jp/kegg/). Enrichment analysis was performed using Bioconductor’s topGO package to investigate the functional categories of DEGs, and the number of DEGs included in each GO term was counted. The relevance of the enriched categories was determined using a classical Fisher test, and the top 500 categories were selected. The map of enriched GO relationships was elaborated considering the first 10 significant nodes.

### 4.8. Construction of Gene Functional Interaction Network

Gene set enrichment analysis (GSEA) was performed from the ranked list of each approach to construct a gene network (based on GO annotation terms extracted from the Gene Ontology Consortium (http://www.geneontology.org)) in order to reveal the interaction of selected genes for which the Search Tool for the Retrieval of Interacting Genes (STRING; https://string-db.org) was used.

### 4.9. Validation Assay Through Real-Time qPCR

Among the differentially expressed genes identified through microarray and molecular network analysis, four of them were selected as candidates for validation assay by RT-qPCR analysis. A frozen aliquot of HCAEC cultures from each experiment was used for microarray analysis to validate the differential expression results. Briefly, the total RNA from each sample was reverse transcribed into cDNA using reverse transcriptase, and the resulting cDNA was then used as a template for quantitative PCR amplification. The amount of cDNA was homogenized at a concentration of 50 ng/µL to facilitate methodology and analysis. The expression levels of candidate genes were measured using the appropriate design, and all tests were performed on the StepOne ™ Real-time PCR system (Applied Biosystems, Fosters City, CA, USA). Quantitative fluorescence data were analyzed using sequence detection software (SDS version 2.2, PE Applied Biosystems). The cycle number at which the amplification plot crossed the threshold was calculated, and the cycle threshold (Ct) values were recorded. The relative expression level of target genes was calculated with the 2^−ΔΔCt^ method [57]. GAPDH was used as a housekeeping gene to normalize RNA amounts. The primer sequences used in this study were as follows: STC1 (F: 5′-GTGACACAGATGGGATGTATGA-3′, R: 5′-TTTAAGCTCTCTTTGACGAATGC-3′); DDIT3 (F: 5′-AGCTGAGTCATTGCCTTTCTC-3′, R: 5′-ACCTCTTGCAGGTCCTCATA-3′); EEIG1 (F:5′-CTGCAGCTGACGTGTAAGG-3′, R: 5′-AGCCCTGAGCTGAGAATAGT-3′); PLA2G4C (F: 5′-AAGGAAAGGCTCACTCAGTAAC-3′, R:5′-CCACCGTGTTTGTACAGGAA-3′); GAPDH (F: 5′-GTGCTGAGTATGTCGTGGAG-3′, R: 5′-GTCTTCTGAGTGGCAGTGAT-3′). The PCR cycling conditions were as follows: 40 cycles of 30 s at 96 °C, 30 s at 60 °C (all primers had the same melting temperature), and 30 s at 72 °C.

## 5. Conclusions

In this experimental study, we observed that mediators present in the serum of patients uremic with MI might be able to affect endothelial function by modifying their gene expression profile, which can contribute to CVD in ESRD patients. We believe that with these preliminary data, subsequent studies could be carried out (safeguarding all the limitations inherent in this in vitro study) that could help to clarify the participation of some identified DEGs.

## Figures and Tables

**Table 3:** Characteristics of the selected patients who provided blood samples.

Characteristic	Description	UWOI Group (*n* = 3)	UWI Group (*n* = 3)
Demographic	Age (years)	66 ± 21	66 ± 11
	Female/male	1/2	1/2
Cause of end-stage renal disease (%)	Diabetic nephropathy	1 (33)	3 (100)
	Arterial hypertension	1 (33)	3 (100)
	Coronary heart disease	0 (0)	3 (100)
	Other or unknown	1 (33)	1 (33)
Comorbidity (%)	Ischemic heart disease	0 (0)	3 (100)
	Myocardial infarction	0 (0)	3 (100)
	History of hypertension	3 (100)	3 (100)
	Mellitus diabetes	3 (100)	3 (100)
	Smoking	2 (67)	0 (0)
	Familial hyperlipidemia	1 (33)	1 (33)
	Familial hypercholesterolemia	1 (33)	1 (33)
Treatment (%)	Insulin	0 (0)	2 (67)
	Folic acid	0 (0)	1 (33)
	Complex B	0 (0)	2 (67)
	Calcitriol	0 (0)	1 (33)
	Enalapril	1 (33)	0 (0)
	Losartan	1 (33)	1 (33)
	Amlodipine	1 (33)	0 (0)
	Clopidogrel	0 (0)	1 (33)
	Acetylsalicylic acid	0 (0)	1 (33)
	Isosorbide	2 (67)	1 (33)
	Statins	0 (0)	1 (33)
Renal replacement therapy	Peritoneal dialysis (%)	3 (100)	0 (0)
	Hemodialysis (2–3 sessions/week)	0 (0)	3 (100)
Biochemical parameters (mg/dl)	Glucose	125 (89–167)	124 (86–189)
	Urea	81 (32–108)	122 (104–134)
	Creatinine	10.5 (1.1–18)	10.6 (5.8–16.1)
	Cholesterol	170 (147–192)	166 (149–192)
	Triglycerides	115 (100–138)	180 (115–223)
	HDL-cholesterol	43 (28–64)	29 (27–32)
	LDL-cholesterol	110 (99–126)	105 (100–112)

UWOI, uremia without infarction; UWI, uremia with infarction; values express *n* (%), mean (range).

## Data Availability

Data are contained within the article.
